# Genomic Disorders: Molecular Mechanisms for Rearrangements and Conveyed Phenotypes

**DOI:** 10.1371/journal.pgen.0010049

**Published:** 2005-12-30

**Authors:** James R Lupski, Pawel Stankiewicz

## Abstract

Rearrangements of our genome can be responsible for inherited as well as sporadic traits. The analyses of chromosome breakpoints in the proximal short arm of Chromosome 17 (17p) reveal nonallelic homologous recombination (NAHR) as a major mechanism for recurrent rearrangements whereas nonhomologous end-joining (NHEJ) can be responsible for many of the nonrecurrent rearrangements. Genome architectural features consisting of low-copy repeats (LCRs), or segmental duplications, can stimulate and mediate NAHR, and there are hotspots for the crossovers within the LCRs. Rearrangements introduce variation into our genome for selection to act upon and as such serve an evolutionary function analogous to base pair changes. Genomic rearrangements may cause Mendelian diseases, produce complex traits such as behaviors, or represent benign polymorphic changes. The mechanisms by which rearrangements convey phenotypes are diverse and include gene dosage, gene interruption, generation of a fusion gene, position effects, unmasking of recessive coding region mutations (single nucleotide polymorphisms, SNPs, in coding DNA) or other functional SNPs, and perhaps by effects on transvection.

## Introduction

Whereas Watson–Crick DNA base pair changes have long been recognized as a mechanism for mutation, rearrangements of the human genome including deletions, duplications, and inversions have been appreciated only more recently as a significant source for genetic variation. Deletion and duplication mutations can vary in size from thousands to hundreds of thousands of base pairs in length and may require specialized technologies to visualize. Structural features, or the architecture, of the human genome can result in region-specific susceptibility to rearrangements and thus genomic instability. The molecular mechanisms by which rearrangement mutations of the human genome occur, and how such rearrangements convey phenotypes, are only beginning to be unraveled.

During the last decade it has become apparent that the molecular genetic mechanisms for many disease traits consist of genomic rearrangements rather than point mutations of single genes. Such conditions, in which the clinical phenotype is a consequence of abnormal dosage or dysregulation of one or more genes resulting from rearrangement of the genome, have been referred to as genomic disorders [1–4]. DNA rearrangements occur by both homologous and nonhomologous recombination mechanisms; however, homologous recombination (HR) appears to be the predominant pathway underlying recurrent rearrangements of our genome. Regardless of mechanism, structural features of the genome can predispose a particular region to rearrangement. Determining the architectural features that result in the instability of the genomic regions has profound consequences for clinical genetics as new technologies enable high-resolution analysis of the human genome. This review will focus on the information culled from, and molecular mechanisms elucidated by, breakpoint analyses of disease-associated rearrangements involving proximal 17p. Although the focus is 17p, such mechanisms appear to be generally applicable to all regions of the human genome. We also describe the many mechanisms by which rearrangements can convey phenotypes and discuss rearrangements as the basis for introducing variation in our genome.

## Proximal 17p Dosage Changes Convey Phenotypes—An Assay for Rearrangements

Charcot-Marie-Tooth disease type 1A (CMT1A) and hereditary neuropathy with liability to pressure palsies (HNPP) are dysmyelinating peripheral neuropathies that result from an altered dosage of *PMP22,* which encodes peripheral myelin protein. CMT1A results from heterozygous duplication of a 1.4-Mb segment that includes the *PMP22* gene, whereas HNPP results from a heterozygous deletion of the same genomic interval. The rearrangements cause altered dosage of *PMP22* that subsequently results in neuropathy; overexpression causes CMT1A whereas underexpression (i.e., haploinsufficiency) leads to HNPP. Experimental evidence in support of the *PMP22* dosage hypothesis is substantive (reviewed in [5,6]). Suffice it to say that rare nonduplication CMT1A patients have been identified with heterozygous apparent gain-of-function *PMP22* point mutations, and rare nondeletion HNPP patients have loss-of-function *PMP22* mutations (nonsense or frameshift alleles) consistent with haploinsufficiency [5]. Animal models that overexpress *PMP22* recapitulate the CMT1A phenotype, and the neuropathy can be clinically, electrophysiologically, and neuropathologically corrected by abrogation of the overexpression using epigenetic manipulation of *PMP22* gene expression [7,8].

Smith-Magenis syndrome (SMS) is a multiple congenital anomaly/mental retardation disorder usually associated with a cytogenetically visible heterozygous deletion of sub-band 17p11.2, i.e., del(17)(p11.2p11.2) (reviewed in [9,10]). Rare patients without deletion have been identified, and some were found to have heterozygous point mutations in the *retinoic acid inducible 1 (RAI1)* gene [11–13]. As would be anticipated, most of these are frameshift or nonsense mutations consistent with a haploinsufficiency mechanism. Chromosome-engineered mouse models that delete one copy of the mouse Chromosome 11 region syntenic to human 17p11.2 (i.e., *Df(11)17* and other derivative deficiencies) [14–16], as well as targeted disruption of *Rai1* [17], recapitulate much of the SMS phenotype. Animal models that are compound heterozygotes for deletion and duplication (*Df(11)17/Dp(11)17*) have a normal phenotype; this “rescue” is consistent with a dosage mechanism for the phenotypes observed in the mice harboring heterozygous rearrangements [14]. A syndrome associated with heterozygous duplication of the genomic interval deleted in SMS, dup(17)(p11.2p11.2), has been described [18]. The dup(17)(p11.2p11.2) phenotype likely results from a dosage-sensitive gene in the human Chromosome 17p11.2 region. This dosage-sensitive gene is probably *RAI1* since *Dp(11)17*/*Rai1* ^−^ animals, who have a normal *Rai1* gene copy number but three copies for all the other genes in the rearranged intervals, have a normal phenotype (i.e., the knockout allele appears to rescue the duplication phenotypes; unpublished data), although this hypothesis awaits formal verification.

Thus, alterations of the copy number of either *PMP22* or *RAI1* convey a clinical phenotype that usually elicits a visit to a physician. Therefore, rearrangements involving these genes can be readily ascertained.

## Recurrent Rearrangement Breakpoints Map to LCRs

**Figure 1:**
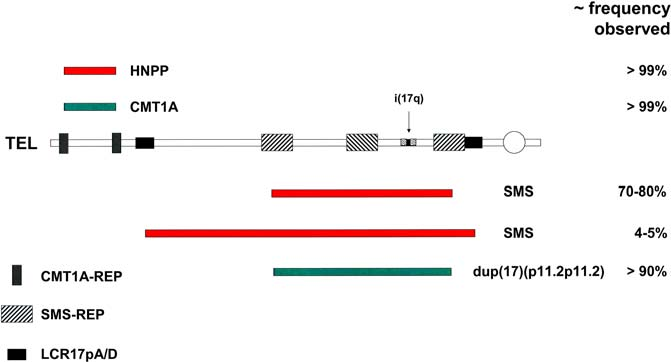
Recurrent Rearrangements in Proximal 17p The horizontal line represents proximal 17p with the telomere (TEL) to the left, the centromere (circle) to the right, and LCRs demarcated. The genomic regions duplicated in CMT1A (green horizontal rectangle) and deleted in HNPP (red horizontal rectangle) are shown above, and the recurrent deletions associated with SMS and duplication associated with dup(17)(p11.2p11.2) are shown below. The position of the isochromosome 17q breakpoint cluster region within a large cruciform structure (consisting of five subunits of ~40–50 kb each) is also shown.

The CMT1A duplication [19] and HNPP deletion [20] are transmitted through the germ line and cosegregate with their respective neuropathy phenotypes as an autosomal dominant trait. However, both de novo duplication and deletion can occur in association with sporadic disease. The vast majority of unrelated patients from families segregating CMT1A, as well as sporadic cases, have the same size duplication. This common duplication rearrangement has recurrent breakpoints that map to LCRs called CMT1A-REPs [21] (Figure 1). Similarly, HNPP patients have a common deletion rearrangement with recurrent (i.e., clustered) breakpoints that map to CMT1A-REPs. It has been shown that the CMT1A duplication and HNPP deletion represent alternative products of a NAHR utilizing CMT1A-REPs as recombination substrates [21,22].

Detection of the CMT1A duplication or HNPP deletion has turned out to be a useful molecular diagnostic test for the evaluation of patients with neuropathy. Thousands of tests that detect a junction fragment (i.e., a novel band that reflects the rearrangement and can be identified at the breakpoint junction) specific to either the duplication or deletion have been performed since the early 1990s. Essentially all CMT1A and HNPP patients with a rearrangement mutation, with the exception of three reported CMT1A patients harboring a smaller duplication and a couple of HNPP patients with smaller deletions (reviewed in [5]), have had the common recurrent rearrangement. Thus, in greater than 99% of the families with rearrangements the new mutation appears to have occurred by NAHR. However, it is important to note that the molecular test that assays for a specific junction fragment may not detect some smaller or larger sized duplications.

In contrast to CMT1A and HNPP, which usually segregate as dominant traits, SMS is essentially always a sporadic disease associated with a de novo del(17)(p11.2p11.2) [23–26]. In the majority of SMS patients with cytogenetically visible deletions, the breakpoints are recurrent and cluster in LCRs termed SMS-REPs [25–28] (Figure 1). The common recurrent SMS deletion occurs by NAHR utilizing SMS-REPs as the recombination substrates [29,30]. A common recurrent rearrangement occurs in 70%–80% of deletion patients with SMS [31].

Approximately 20%–30% of SMS patients do not harbor the common deletion, but instead have uncommon sized deletions. Interestingly, some of the uncommon deletion rearrangements [32,33], representing about 4% of the total SMS deletions studied, were also found to have recurrent breakpoints. As anticipated, these recurrent breakpoints mapped to yet another LCR family—LCR17ps [34] (Figure 1). These uncommon recurrent SMS rearrangements also occur by NAHR, utilizing LCR17p flanking repeats as recombination substrates. Whereas the predicted reciprocal duplication of the common SMS deletion mediated by SMS-REP has been identified [18], the predicted reciprocal duplication for this uncommon recurrent deletion remains to be found.

## Recombination Hotspots Associated with Strand Exchanges

Theoretically, HR can occur whenever there is a shared stretch of homology providing substrates. There does appear to be a minimal stretch of identity, referred to as a minimal efficient processing segment (MEPS), required among substrates to enable HR to occur. The MEPSs that enable HR to occur in cultured mouse cells have been determined to be between 132 and 232 bp of perfect shared sequence identity [35,36]. The MEPS requirements for HR in human meiosis remain to be elucidated. Nevertheless, for an LCR of several thousand base pairs in length and more than 98% identity, a strand exchange could occur potentially wherever there are the required MEPSs. However, experimental observations from multiple NAHR studies document positional preferences, or recombination hotspots, wherein the crossovers preferentially occur [37]. This was initially observed within the 24-kb CMT1A-REP [37,38], but found also in the ~200-kb SMS-REP [30] and ~125-kb LCR17p [34]. Interestingly, hotspots for strand exchange have been documented also for allelic HR (AHR) across the human genome [39–41]. Common features shared among NAHR and AHR hotspots include the following: clustering within small (<1 kb) genomic regions, coincidence with apparent gene conversion events, and no obvious sequence similarities with one another [37]. This last feature distinguishes mammalian HR from HR in prokaryotes, wherein a *cis*-acting recombinogenic heptameric sequence motif (χ or chi [42]) stimulates recombination. Whether NAHR and AHR hotspots are coincident in the human genome remains to be determined. It is also not clear if recombination hotspots reflect *cis*-acting sequence motifs, positional preference of *trans*-acting factors, or unusual non-B DNA structures [43], or rather just denote genomic regions more susceptible to DNA double-strand breaks.

## NAHR—A General Mechanism for Generating Rearrangements of Our Genome

With the description of the reciprocity for NAHR, e.g., the CMT1A duplication/HNPP deletion and the SMS deletion/dup(17)(p11.2p11.2), it is anticipated that all deletion syndromes in which the rearrangement breakpoints cluster in flanking LCRs will likely have reciprocal duplication syndromes. One challenge is to identify such reciprocal duplications and document their role in causing a specific phenotype. In addition to deletion/duplication rearrangements mediated by NAHR using directly oriented LCRs as substrates, NAHR can also produce inversion rearrangements if inverted LCRs are utilized as the recombination substrates. Such inversion rearrangements can disrupt genes and cause disease traits [44], predispose DNA to deleterious genomic rearrangements [45–48], or be responsible for haplotype blocks essentially creating a balancer chromosome that suppresses recombination [49]. Somatic NAHR between nonsister chromatids can result in the formation of an isochromosome [50].

## Nonrecurrent Rearrangements

**Figure 2:**
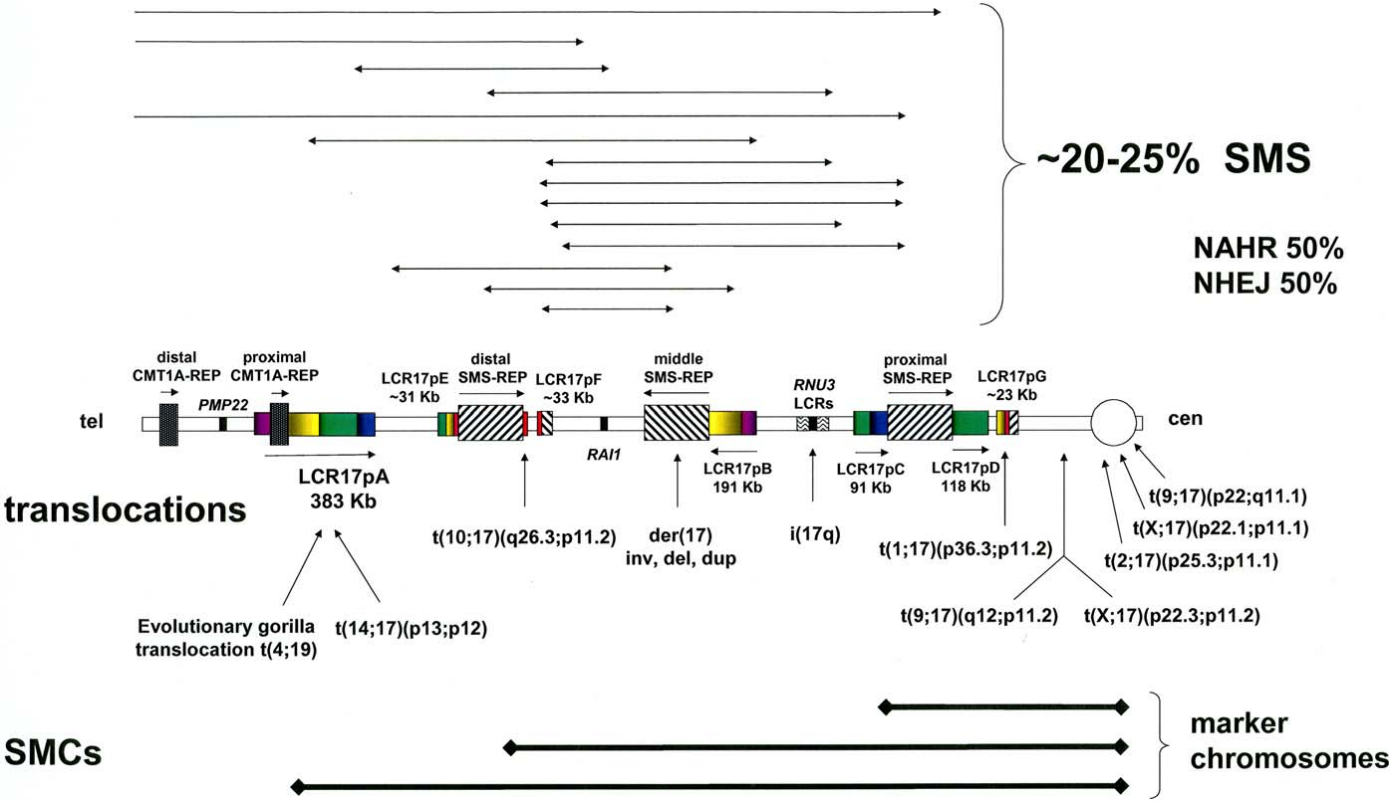
Nonrecurrent Rearrangements in Proximal 17p Proximal 17p with its complex genome architecture and multiple LCRs. The centromere (cen) is to the right, telomere (tel) to the left. Filled, hatch-marked, and color-coded rectangles depict LCR regions of greater than 97% sequence identity, with horizontal arrows depicting orientation. The locations of the *RAI1* gene and isochromosome 17q breakpoint cluster regions are demarcated. Above is shown the region deleted in SMS patients with uncommon nonrecurrent deletions—the breakpoints are denoted by arrowheads. Below are shown the regions contained in the supernumerary marker chromosomes (SMCs). Also, below are shown the 17p11.2 breakpoints of the translocations.

The breakpoints of ~20%–30% of deletions in patients with SMS do not map to the proximal and distal copies of SMS-REP as in the common recurrent deletions [32,33] (Figure 2). Such deletion patients are readily ascertained because their phenotype also results from *RAI1* haploinsufficiency. Interestingly, the breakpoints of these nonrecurrent rearrangements often map to LCRs [33]. However, the observation that the two breakpoints could be in different LCRs is inconsistent with homology mediating these events. Thus, LCRs may stimulate but do not appear to mediate nonrecurrent rearrangements. Sequencing the breakpoint junctions to examine the products of recombination for four such nonrecurrent rearrangements revealed NHEJ as the mechanism in two whereas the other two represented *Alu*–*Alu* recombinations between closely related (i.e., sharing a high degree of sequence identity) *Alu* sequences [51].

LCRs have also been identified at the breakpoints of three of four small marker Chromosomes 17 [52–54] and in some apparently balanced translocations with breakpoints in 17p [33] (Figure 2), but the DNA sequence at these breakpoints has not been determined so the exact recombination mechanism remains to be elucidated. Interestingly, breakpoints for small marker chromosomes and translocations also often map to (peri)centromeric sequences.

## NHEJ—An Alternative Pathway

It is clear that not all rearrangements in our genome are mediated by HR. As documented above, evidence for NHEJ has been found by examining breakpoints for some deletions causing SMS. However, this represents less than 20%–25% of SMS deletion cases. Nevertheless, it remains to be determined to what extent NHEJ is a mechanism for genome rearrangement. NHEJ may potentially have a more prominent role in nonrecurrent rearrangements [55–57].

## Somatic Rearrangements

The molecular investigations of somatic rearrangements pose additional challenges to those encountered in the study of constitutional rearrangements. In constitutional rearrangements the tissue used for a source of DNA is usually uniform in its genetic constitution. In a somatic rearrangement event, the tissue source for isolating the DNA to study by molecular methods may represent a mosaic mixture of cells that contain the rearrangement with cells that have a normal, or wild-type, genome. This may be further complicated in a tumor, wherein multiple different and serial rearrangement events can occur. Nevertheless, for one somatic 17p rearrangement, molecular analyses revealed complex genomic architecture at clustered breakpoints and led to a model that explains the molecular mechanism for its formation [50].

Isochromosome 17q is a common recurrent genomic rearrangement observed in human neoplasms and was shown earlier to be isodicentric with clustered breakpoints [58]. Subsequently, a complex genomic architecture characterized by large (38–49 kb) cruciform LCRs was identified at the breakpoint cluster region [50]. DNA breaks generated in the hairpin/cruciform structures were postulated to trigger the double-strand-break repair pathway. A subsequent NAHR event between repeats of opposite orientation on sister chromatids (i.e., sister chromatid exchange) can result in the formation of an isodicentric Chromosome 17 and an acentric fragment [50]. The recognition of breakpoint clustering and determination of the mechanism for isochromosome formation enabled the development of a FISH-based test to assay the rearrangement event [59].

## Molecular Mechanisms by Which Constitutional Rearrangements Convey Phenotypes

**Figure 3:**
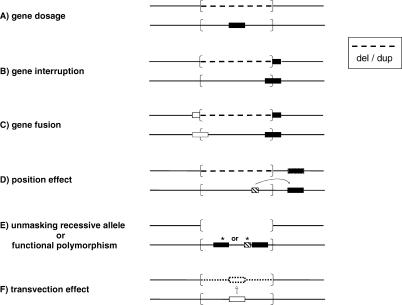
Molecular Mechanisms for Genomic Disorders Six models are depicted and include (A) gene dosage, where there is a dosage sensitive gene within the rearrangement; (B) gene interruption, wherein the rearrangement breakpoint interrupts a gene; (C) gene fusion whereby a fusion gene is created at the breakpoint that either fuses coding sequences or a novel regulatory sequence to the gene; (D) position effect, in which the rearrangement has effects on expression/regulation of a gene near the breakpoint, potentially by removing or altering a regulatory sequence; (E) unmasking recessive allele, where a deletion results in hemizygous expression of a recessive mutation or further uncovers/exacerbates effects of a functional polymorphism; and (F) by potentially interrupting effects of transvection, where the deletion of a gene and its surrounding regulatory sequences affects the communication between alleles. In each model, both chromosome homologs are depicted as horizontal lines. The rearranged genomic interval is enclosed by brackets—dashed lines indicate genomic regions either deleted or duplicated, an absent line indicates deletion with phenotypic effects from the remaining allele unmasked because of the rearrangement, and a dotted line represents deletion but where phenotypic effects result from the absence of interactions between alleles (i.e., transvection effects). Gene is depicted by filled horizontal rectangle, while regulatory region is shown as a hatch-marked rectangle. Asterisks denote point mutations.

Deletion and duplication rearrangements can cause a phenotype by several molecular mechanisms (Figure 3A–3D), including altering the copy number of a gene (or genes) sensitive to a dosage effect, as exemplified by *PMP22* and *RAI1*. The breakpoint of the rearrangement may interrupt a gene and cause a loss-of-function by inactivating a gene. Alternatively, a fusion gene can form at the breakpoint generating a gain-of-function mutation; a mechanism prominent amongst cancers associated with specific chromosomal translocations. Rearrangements can also manifest through a position effect [60]. Such position effects have been documented for apparently balanced translocations that even exert their influence when the breakpoints map as far as ~1 Mb away either upstream or downstream from the culprit gene [61]. Position effects have been observed also with deletion [60] and duplication [62] rearrangements that occur outside the intact gene.

Other molecular mechanisms by which rearrangements of the genome may convey or alter a disease phenotype result from how the rearrangement on one chromosome affects or is affected by the allele on the other chromosome at that locus (Figure 3E and 3F). These include the unmasking of either recessive mutations (reviewed in [63]) or functional polymorphisms [64] of the remaining allele when a deletion occurs, and potential transvection (communication between alleles on homologous chromosomes) [16,17] effects via deletion of regulatory elements required for communication between alleles.

## Copy-Number Variations

Recent excitement has been generated by the observation that individuals may vary for large segments of their genome, with evidence for both decreased and increased copy number [65–67]. This revelation has been enabled by array technologies that allow high-resolution screening of the entire human genome simultaneously. It is not clear to what extent such genomic changes are responsible for Mendelian or complex disease traits and common traits (including behavioral traits), or represent only benign polymorphic variation. In fact, it is impossible to assay individuals with such genomic changes for all potential phenotypes that can occur. Furthermore, some phenotypes caused by genomic rearrangements (e.g., HNPP) may not present until late adulthood—if at all [5,6]. This age-dependent penetrance confounds the interpretation of genomic copy-number changes. Copy-number changes have been associated with phenotypes that are often difficult to ascertain such as susceptibility to HIV infection [68].

**Figure 4:**
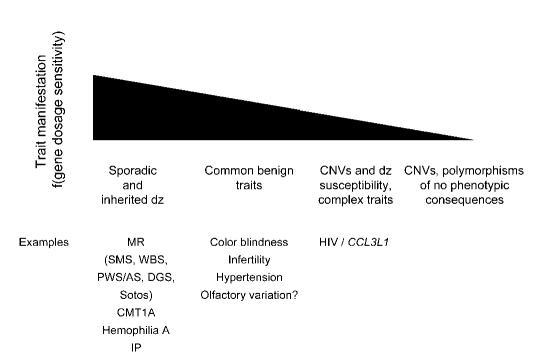
Genomic Rearrangements and Phenotypic Traits Above is shown a gradient/threshold for trait manifestation. Whether or not a trait is manifested is a function of the dosage sensitivity of the gene(s) affected by the rearrangement. Below are examples of traits that can be due to DNA rearrangements. DGS, DiGeorge syndrome; dz, disease; IP, incontientia pigmenti; MR, mental retardation; PWS/AS, Prader-Willi syndrome/Angelman syndrome; WBS, Williams-Beuren syndrome.

**Figure 5:**
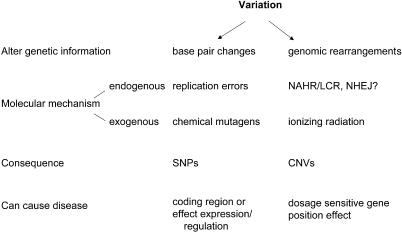
CNVs versus Nucleotide Changes The two major mechanisms by which variation is introduced into our genome are shown. Such variations can be introduced by both endogenous and exogenous means. These mutations can cause a disease trait if they affect gene structure, function, or regulation, as well as through the alteration of dosage. SNP, single nucleotide polymorphism.

Copy-number variations (CNVs), alternatively referred to as large-segment copy-number variations (LCVs) [65] or copy-number polymorphisms (CNPs) [66], of genomic regions have been reported to occur near segmental duplications or LCRs [65,66,69]. However, the involvement of segmental duplications, perhaps by an LCR/NAHR mechanism, is yet to be determined. Segmental duplications account for some 5%–10% of the human genome [70–72], and CNVs may be coincident with LCRs by chance. Nevertheless, it is clear that LCR/NAHR-generated rearrangements occur throughout the genome [1,2], and therefore it is not unreasonable to assume that such rearrangements or CNVs could be associated with inherited or sporadic (de novo rearrangement) disease, susceptibility to disease, complex traits, or common benign traits, or could represent polymorphic variation with no apparent phenotypic consequences (Figure 4), depending on whether or not dosage-sensitive genes are affected by the rearrangement. In fact, analogous to base pair changes, rearrangements introduce variations into the genome for selection to act upon (Figure 5). Perhaps LCR/NAHR is analogous to the changes introduced by a replication error at a nucleotide base: both are endogenous molecular mechanisms that introduce variation into our genome. Early comparative genomics studies among bacterial species revealed substantive evidence for genome rearrangements and insertion/deletion events that accompany genome evolution [73,74].

## Conclusion

During the previous decade, we have witnessed the uncovering of recurrent submicroscopic rearrangements as a cause of disease. High-resolution analysis of the human genome has allowed detection of genome changes not observed previously because of technology limitations [4]. The availability of the “finished” human genome sequence [75] and genomic microarrays have enabled approaches to resolve changes in the genome heretofore impossible to assess, particularly on a global genome scale, i.e., simultaneously examining the entire genome rather than discreet segments [76]. During the past five decades, since the elucidation of the chemical basis of heredity by Watson and Crick, base pair changes have dominated our thinking with regard to mutation and variation. Rearrangements of our genome are perhaps introducing mutation and variation to a greater extent than was recognized previously. 

## Abbreviations

17p: proximal short arm of Chromosome 17
AHR: allelic homologous recombination
CMT1A: Charcot-Marie-Tooth disease type 1A
CNP: copy-number polymorphism
CNV: copy number variation
HNPP: hereditary neuropathy with liability to pressure palsies
HR: homologous recombination
LCR: low-copy repeat
LCV: large-segment copy-number variation
MEPS: minimal efficient processing segment
NAHR: nonallelic homologous recombination
NHEJ: nonhomologous end-joining
*RAI1,*: *retinoic acid inducible 1;*
SMS: Smith-Magenis syndrome
